# Introduction to the special issue related to the 19th International Small Angle Scattering Conference (SAS2024)

**DOI:** 10.1107/S160057672500682X

**Published:** 2025-08-28

**Authors:** U-Ser Jeng, Jan Ilavsky, Elliot Paul Gilbert, Wei-Tsung Chuang, Orion Shih

**Affiliations:** a National Synchrotron Radiation Research Center Hsinchu300092 Taiwan; b Department of Chemical Engineering and College of Semiconductor Research National Tsing Hua University Hsinchu300044 Taiwan; c Advanced Photon Source Argonne National Laboratory 9700 S. Cass Avenue Argonne IL60439 USA; d Australian Centre for Neutron Scattering Australian Nuclear Science and Technology Organisation New Illawarra Road Lucas Heights NSW2234 Australia

**Keywords:** small-angle scattering

## Abstract

The virtual special issue related to the 19th International Small Angle Scattering Conference (SAS2024, Taipei, Taiwan) is introduced. The articles included here were originally published in recent regular issues of *Journal of Applied Crystallography* and *Journal of Synchrotron Radiation*. The SAS2024 special issue is available at https://journals.iucr.org/special_issues/2025/sas2024/.

The 19th International Small Angle Scattering Conference (SAS2024) was hosted by the National Synchrotron Radiation Research Center (NSRRC) and took place from 3 to 8 November 2024 at the Taipei International Convention Center (TICC), Taiwan. Since the first SAS meeting in 1965, the triennial SAS conference has become the flagship event for our community, most recently convening in Campinas in the state of São Paulo, Brazil, for SAS2022 (Meneau *et al.*, 2023 ▸; Trewhella, 2021 ▸). This time, SAS2024 attracted 508 participants from 32 countries and showcased 433 abstracts (240 oral presentations and 193 posters).

Under the leadership of Conference Chair U-Ser Jeng, Scientific Program Committee Chair Wei-Tsung Chuang and Local Organizing Committee Chair Orion Shih, together with an outstanding team of NSRRC staff, a dedicated Scientific Program Committee and an engaged International Advisory Committee, SAS2024 delivered a comprehensive scientific program. Topics spanned bioSAS, polymers and composites, imaging with SAS and coherent diffraction, magnetic and condensed matter systems, soft matter, colloids, gels and complex fluids, interfaces and surfaces, kinetics and dynamics, instruments and methodology, modeling and data analysis, and industrial applications. A dedicated session on ‘SAS for Semiconductor Science and Industry’ highlighted Taiwan’s world-leading expertise in this sector.

A memorial session celebrated the life and achievements of the late Academician Professor Sow-Hsin Chen (2015 Guinier Prize winner). Preceding the main conference, a one-day SAS school combined tutorials and lectures delivered by the enthusiastic Paul Butler, Stephen King, Brian Pauw, Jan Skov Pedersen, Daniel Franke and Melissa Graewert. It included demonstrations of the *SasView* and *ATSAS* software packages and attracted more than 80 participants. The school also ran in parallel with the canSAS2024 meeting chaired by Paul Butler and U-Ser Jeng (or canSAS-XIV, with over 120 registrants; https://wiki.cansas.org/index.php?title=canSAS-XIV). In addition, the SAS2024 satellite meeting ‘Cutting-Edge in Soft Matter Science 2024’ (CeSMS2024), organized by Kazuo Sakurai, was held in Kitakyushu, Japan, on 30–31 October 2024 and drew over 70 attendees, including several joint speakers.

More conference highlights included the 2024 Guinier Prize awarding ceremony and lecture given by the winner, Professor Takeji Hashimoto (Kyoto University) (https://www.sas2024.tw/site/page.aspx?pid=569&sid=1535&lang=en). The Otto Kratky Prize in SAS2024 for young scientists of career prospects in SAS was awarded to Grace Causer from Monash University, Australia. Funding from the International Union of Crystallography (IUCr) and Asia–Oceania Forum for Synchrotron Radiation Research (AOFSRR) supported 13 early career researchers and students to attend. Ten poster prizes and one Best Paper award were also presented.

Community-building activities featured an open meeting of the IUCr Commission on SAS and bid presentations for SAS2030, submitted by delegations from Republic of Korea and India. More than 250 participants enjoyed a facility tour of NSRRC’s 3 GeV Taiwan Photon Source and 1.5 GeV Taiwan Light Source, including the three high-performance SAXS–WAXS beamlines equipped with bio­SWAXS, spin-coating and coherent-microbeam sample environments.

The conference banquet at the National Palace Museum’s Silks Palace blended Taiwanese culture with international friendship. Guests were treated to a diabolo performance, folk songs from the ‘NSRRC CAT band’ and a multilingual rendition of *To a Wild Rose*, before the SAS baton—a tradition since SAS2009—was ceremonially passed to the SAS2027 hosts (MAX IV and the European Spallation Source, Sweden). The venue for SAS2030 was also announced as the Pohang Accelerator Laboratory (PAL) and HANARO reactor (KAERI), Republic of Korea.

Continuing the tradition started with SAS2012, selected work presented at the SAS conferences has been published in special issues of *Journal of Applied Crystallography* (*JAC*), with open-access support from the IUCr. In this virtual special issue of SAS2024 (https://journals.iucr.org/special_issues/2025/sas2024/), 47 submissions were received, handled primarily by Guest Editors U-Ser Jeng, Jan Ilavsky and Elliot Gilbert, along with Guest Co-editors Florian Meneau, Dmitri Svergun and Judith Houston, and 31 have been selectively published across volumes 57 (2024) and 58 (2025) of *JAC* and volume 32 (2025) of *Journal of Synchrotron Radiation*. This special issue represents an excellent overview of the current state of SAS applications and instrumentation with anticipated future developments. Two papers—by Lu *et al.* (2024 ▸) on GISAXS–GIWAXS for *in situ* spin-coating and Kang *et al.* (2025*b* ▸) on SAXS–WAXS studies of drug-loaded liposomes—were selected as *JAC* cover articles. Six papers cover major facility instrumentation using X-rays (*e.g.* Ma *et al.*, 2025 ▸) and neutrons (*e.g.* Kang *et al.*, 2025*a* ▸), sample environments (*e.g.* Iwase *et al.*, 2025 ▸), and innovative measurement techniques (*e.g.* Causer, 2025 ▸; Narayanan *et al.*, 2025 ▸), including a SAXS/WAXD beamline with a laboratory SAXS apparatus at the new synchrotron facility NanoTerasu (Meguro *et al.*, 2025 ▸). Additionally, two papers (Ding *et al.*, 2025 ▸; Tung *et al.*, 2025 ▸) illustrate the application of machine learning/artificial intelligence as a tool for enhancing the interpretation of SAS data. The open-source software reports by Brookes *et al.* (2025 ▸) and Franke *et al.* (2025 ▸) highlight recent advancements in widely used bioSAXS tools and are anticipated to attract significant attention, while Larsen *et al.* (2025 ▸), Marcone *et al.* (2025 ▸) and Nishiyama (2025 ▸) describe, respectively, web-based tutorials for the teaching of SAS data analysis, advanced form factors of prismatic particles and structure factors for random hard disk packing in 2D. Importantly, numerous papers showcase new scientific contributions of SAS to our traditional fields, such as bioSAXS (*e.g.* Kang *et al.*, 2025*b* ▸; Shih *et al.*, 2025 ▸) and polymers (*e.g.* Vo *et al.*, 2025 ▸; Striker *et al.*, 2025 ▸; Lin *et al.*, 2025 ▸). The organizing committee gratefully acknowledges all submitting authors and reviewers for their contributions and thanks the staff of the IUCr Editorial Office (Chester, UK) and Editor-in-chief Andrew J. Allen for their unwavering support. Together with the published SAS2024 scientific program (https://www.sas2024.tw/site/page.aspx?pid=587&sid=1535&lang=en), this special issue provides a comprehensive perspective on the current landscape and future trends in the field of small-angle scattering.

The conference Chairs sincerely thank the Chair and Vice-chair of the IUCr Commission on Small-Angle Scattering, Jan Ilavsky and Elliot Gilbert, for their indispensable guidance from the earliest planning stages to publication. Appreciation extends to the administrative secretaries Rung-An Liu, Ya-Wen Chang and Yen-Yin Chen, as well as Project Director Ting Yang and her dedicated conference-organizing team for their remarkable professionalism. Generous support from the National Science and Technology Council (NSTC), Taipei City Government, MFA, IUCr, AOFSRR, FCDD-AP, NSRRC and TWNSS ensured the sustainability and impact of SAS2024. Commercial sponsors went beyond traditional roles and activities by actively contributing to the scientific sessions and deepening their engagement with the SAS community.

Looking ahead, we wish the organizers of SAS2027 in Lund and SAS2030 in Gyeongju every success in carrying forward the rich legacy of the SAS conference series.
